# Comparative Evaluation of the Antimicrobial Properties of Glass Ionomer Cements with and without Chlorhexidine Gluconate

**DOI:** 10.5005/jp-journals-10005-1342

**Published:** 2016-06-15

**Authors:** Josna Vinutha Yadiki, Sharada Reddy Jampanapalli, Suhasini Konda, Hema Chandrika Inguva, Vamsi Krishna Chimata

**Affiliations:** 1 Assistant Professor, Department of Pedodontics and Preventive Dentistry, Army College of Dental Sciences, Secunderabad, Telangana, India; 2 Professor and Head, Department of Pedodontics and Preventive Dentistry Government Dental College and Hospital, Hyderabad Telangana, India; 3 Associate Professor, Department of Pedodontics and Preventive Dentistry Government Dental College and Hospital, Hyderabad Telangana, India; 4 Assistant Professor, Department of Pedodontics and Preventive Dentistry Government Dental College and Hospital, Hyderabad Telangana, India; 5 Assistant Professor, Department of Pedodontics and Preventive Dentistry, Terna Dental College, Mumbai, Maharashtra, India

**Keywords:** Antimicrobial properties, Chlorhexidine gluconate, Glass ionomer cements.

## Abstract

**Background:** Chlorhexidine gluconate is a widely used antimicrobial agent. Adding chlorhexidine and quaternary ammonium compounds to filling materials, such as composite resins, acrylic resins, and glass ionomer cements increases the antibacterial property of restorative materials. This study includes antibacterial property of glass ionomer restorative cements with chlorhexidine gluconate.

**Aim:** The primary objective of our study was to compare the antimicrobial properties of two commercially available glass ionomer cements with and without chlorhexidine gluconate on strains of mutans streptococci.

**Materials and methods:** Two glass ionomers (Fuji II Conventional and Fuji IX) were used. Chlorhexidine gluconate was mixed with glass ionomer cements, and antimicrobial properties against mutans streptococci were assessed by agar diffusion. The tested bacterial strain was inhibited and the antimicrobial properties decreased with time.

**Results:** The highest amount of antimicrobial activity with mean inhibitory zone was found in Fuji II with chlorhexidine gluconate followed by Fuji IX with chlorhexidine gluconate, Fuji II without chlorhexidine gluconate, and Fuji IX without chlorhexidine gluconate.

**Conclusion:** The results of the study confirmed that the addition of 5% chlorhexidine gluconate to Fuji II and Fuji IX glass ionomer cements resulted in a restorative material that had increased antimicrobial properties over the conventional glass ionomer cements alone for *Streptococcus mutans.*

**How to cite this article:** Yadiki JV, Jampanapalli SR , Konda S, Inguva HC, Chimata VK. Comparative Evaluation of the Antimicrobial Properties of Glass Ionomer Cements with and without Chlorhexidine Gluconate. Int J Clin Pediatr Dent 2016;9(2):99-103.

## INTRODUCTION

Caries disease still remains a major public health problem despite the widespread use of fluoride and the decline in caries prevalence observed in the majority of highly industrialized countries.^1^ Because of low socioeconomic status, caries epidemiology still remains an indispensible part of dental public health leading to an increase in caries prevalence.

Seppa et al^2^ reported that glass ionomers have antibacterial properties *in vitro.* Also, the growth of *Streptococcus mutans* has been reported to be inhibited *in vivo* around conventional and silver glass ionomers, which has generally been attributed to fluoride released by the materials.^3^

There may be direct correlation between fluoride release and antimicrobial effects of glass ionomer cements.

Recently, researchers modified filling materials, such as composite resins, acrylic resins, and glass ionomer cements by adding chlorhexidine and quaternary ammonium compounds.^4^ Moreover, bacteriostatic and bactericidal agents have the potential to be used in combination with glass ionomer cements to obtain an antibacterial restorative material.

Chlorhexidine gluconate is a widely used antimicrobial agent as it is both inhibitory and lethal to vegetative Gram-positive and Gram-negative bacteria at relatively high dilutions.^5^ Major characteristics of chlorhexidine that contribute to its success as an antiplaque agent include its substantivity and broad spectrum of antibacterial activity.^6^ It creates bacteriostatic environment by binding to oral surfaces and subsequent release of the compound over a long period of time. Our study was aimed to evaluate the antimicrobial properties of glass ionomer materials (Fuji II and Fuji IX) with and without chlohexidine gluconate against *S. mutans.*

**Fig. 1:**
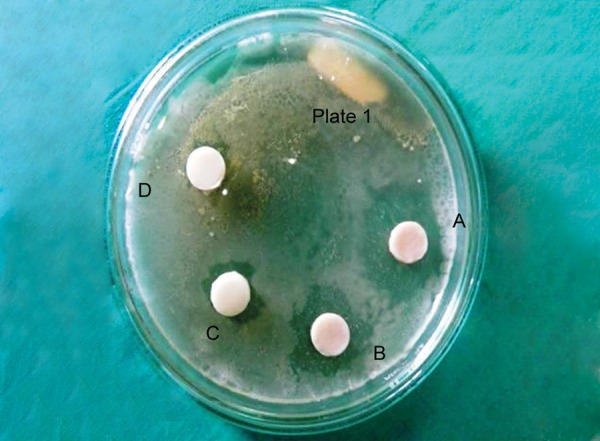
Zones of inhibition around restorative materials against *Streptococcus mutans* on day 1

**Fig. 2:**
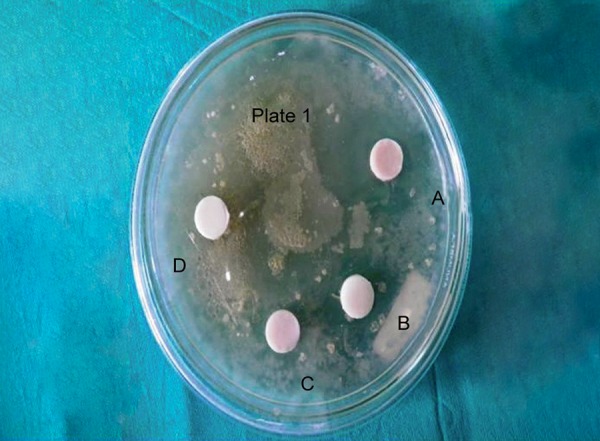
Zones of inhibition around restorative materials against *Streptococcus mutans* on day 7

## MATERIALS AND METHODS

Materials used were Fuji II conventional (Type II GIC), Fuji IX (Type IX GIC; G.C. Corporation, Tokyo, Japan), and 5% chlorhexidine gluconate solution (Basic Pharma Life Sciences Pvt. Ltd, Ankleshwar, Gujarat).

### Method of Preparation of Material Disks

Fuji II with chlorhexidine gluconate, Fuji IX with chlorhexidine gluconate, Fuji II without chlorhexidine gluconate, and Fuji IX without chlorhexidine gluconate were indicated with groups A to D respectively. A total of 10 specimens were prepared from each material.

Specimens of four groups of cements were prepared by using a brass mold containing four holes measuring 11 × 5 mm diameter. 0.5 gm of chlorhexidine gluconate was added to 9.5 gm of liquid glass ionomer cements to obtain 5% formulation. Recommended powder/liquid ratio for restorative purposes by the manufacturers was adopted, i.e., 1 scoop of powder to 1 drop of liquid.

Materials were mixed and loaded into the specific holes in brass mold. Prepared specimens were inserted in the wells within 1 minute with sterile dental instruments. Surface was covered with glass slide and materials were allowed to set. After setting, the disk-shaped specimens were removed from the mold.

### Agar Diffusion Assay

Standard strains of *S. mutans* (MTCC 497) were used to test the antimicrobial efficacy of two different restorative materials with and without chlorhexidine gluco-nate. Brain heart infusion broth was used for culture. Ten agar plates were used. Using a sterile swab, the surface of each agar plate was swabbed three times to ensure even distribution of the inoculum. After drying of the agar plates, four wells of 11 × 5 mm diameter were made in each agar plate using sterile agar punchers and the set disk-shaped specimens were inserted into the wells. The plates wee incubated aerobically for 48 hours.

The experiment was repeated ten times for each material and the zones of inhibition were measured independently. Mean zone of inhibition of each restorative material was calculated and subjected to statistical analysis.

## RESULTS

**Fig. 3:**
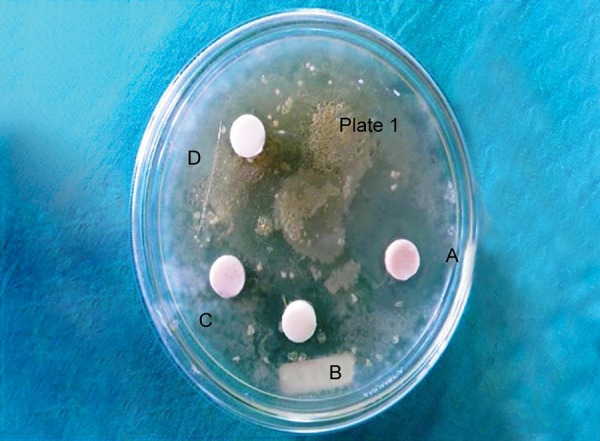
Zones of inhibition around restorative materials against *Streptococcus mutans* on day 14

Antimicrobial activity was determined by measuring the size of inhibition zones produced around the specimens (specimen + inhibition zone, in mm) with a digital caliper at three different points, and then the mean was recorded after 24 hours to obtain day 1 values (Fig. 1). This procedure was continued to obtain day 7 (Fig. 2) and day 14 (Fig. 3) values.

**Graph 1:**
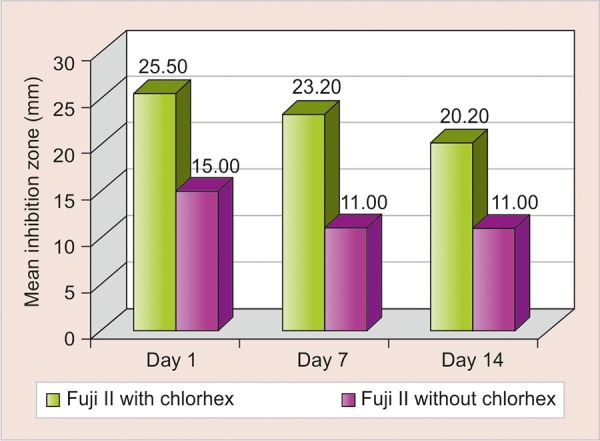
Comparison of mean inhibition zone of Fuji II with chlorhexidine gluconate *vs* Fuji II without chlorhexidine gluconate

**Graph 2:**
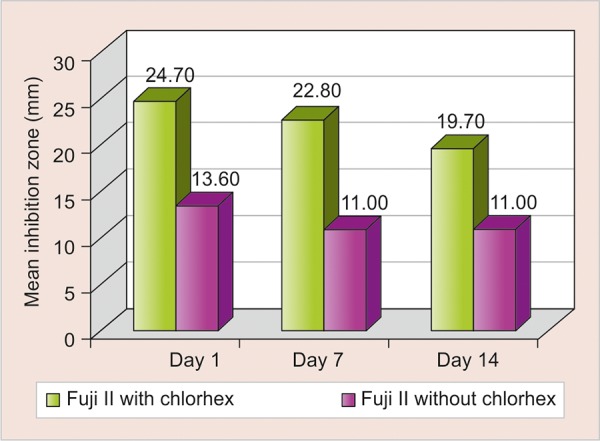
Comparison of mean inhibition zone of Fuji IX with chlorhexidine gluconate *vs* Fuji IX without chlorhexidine gluconate

All four groups of restorative materials showed antimicrobial properties. *Streptococcus mutans* was inhibited and the antimicrobial properties decreased with time.

The mean inhibition zone of Fuji II with chlorhexidine gluconate *vs* Fuji II without chlorhexidine gluconate (Graph 1) and mean inhibition zone of Fuji IX with chlorhexidine gluconate *vs* Fuji IX without chlorhexidine gluconate (Graph 2) were compared.

The highest amount of antimicrobial activity with mean inhibitory zone was found in Fuji II with chlorhexidine gluconate (25.50 ± 1.269) followed by Fuji IX with chlorhexidine gluconate (24.70 ± 0.949), Fuji II without chlorhexidine gluconate (15.00 ± 0.667), and Fuji IX without chlorhexidine gluconate (13.60 ± 0.516) on day 1 (Table 1).

**Graph 3:**
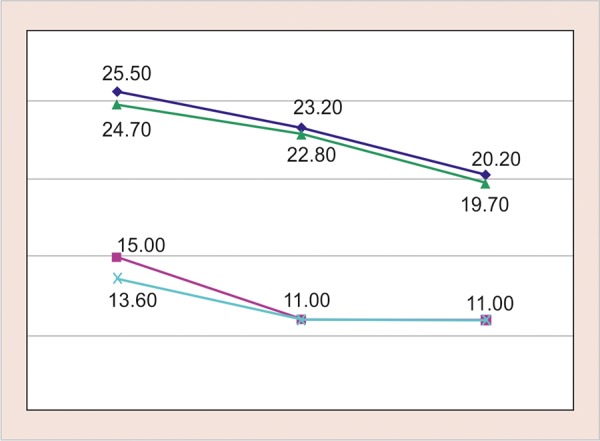
Mean inhibition zone in four groups on days 1, 7, and 14

Tukey HSD *post hoc* analysis showed that the mean inhibition in both Fuji II and Fuji IX with chlorhexidine gluconate was significantly greater than that of Fuji II and Fuji IX without chlorhexidine gluconate up to 14 days. One-way analysis of variance showed that the difference in mean inhibition zone was statistically highly significantly between four groups (p < 0.001) up to 14 days (Table 2 and Graph 3).

**Table 1:** **Table 1:** Mean values of inhibition zones (mm) of four groups of restorative materials against *Streptococcus mutans*

		*Mean values of* *inhibition zones*						*Standard deviation*						*Analysis of* *variance*	
*Materials*		*Day 1*		*Day 7*		*Day 14*		*Day 1*		*Day 7*		*Day 14*		*(Sig)*	
Group A		25.50		23.20		20.20		1.269		1.229		1.317		<0.001*	
Group B		24.70		22.80		19.70		0.949		0.632		1.252			
Group C		15.00		11.00		11.00		0.667		0.000		0.000			
Group D		13.60		11.00		11.00		0.516		0.000		0.000			

*Highly significant (p < 0.001)

**Table 2:** **Table 2:** Comparison of intragroup change in inhibition zone from day 1 to 7 and then at day 14

*Materials*		*Comparison*		*Mean* *difference*		*“t”-value*		*p-value*	
Group A		Day 1 *vs* day 7		2.30 ± 0.68		10.776		<0.001*	
		Day 1 *vs* day 14		5.30 ± 1.06		15.821		<0.001*	
		Day 7 *vs* day 14		3.00 ± 0.67		14.230		<0.001*	
Group B		Day 1*vs* day 7		1.90 ± 0.74		8.143		<0.001*	
		Day 1 *vs* day 14		5.00 ± 1.25		12.677		<0.001*	
		Day 7 *vs* day 14		3.10 ± 0.99		9.858		<0.001*	
Group C		Day 1 *vs* day 7		4.00 ± 0.67		18.974		<0.001*	
		Day 1 *vs* day 14		4.00 ± 0.67		18.974		<0.001*	
		Day 7 *vs* day 14		–		–		–	
Group D		Day 1 *vs* day 7		2.60 ± 0.52		15.922		<0.001*	
		Day 1 *vs* day 14		2.60 ± 0.52		15.922		<0.001*	
		Day 7 *vs* day 14		–		–		–	

*Highly significant (p < 0.001)

Group A―Fuji II with chlorhexidine gluconate

Group B―Fuji IX with chlorhexidine gluconate

Group C―Fuji II without chlorhexidine gluconate

Group D―Fuji IX without chlorhexidine gluconate

## DISCUSSION

Our study demonstrated that the addition of chlorhexidine gluconate to Fuji II and Fuji IX glass ionomer cements resulted in a restorative material that had increased antimicrobial properties over the conventional glass ionomer alone for *S. mutans.* There was significant difference in antimicrobial activity of four groups of materials. This antimicrobial activity of the cements may be due to fluoride release.

The mutans group streptococci are the most cari-ogenic microorganisms because of their metabolic characteristics and activity. The risk of developing caries in patients is determined by the level of microorganisms in saliva.

The most attractive advantage of the glass ionomers is their ability to release fluoride in the immediate vicinity of the cement. Fluoride release seems to be the most probable reason for the inhibitory effect on acid production. Fluoride availability from glass ionomer is pH controlled, the rate-controlling factors being salivary phosphate and proteins. Shashibhushan et al^7^ reported that there is a direct correlation between the amount of fluoride release and the antibacterial activity.

Vermeersch^8^ reported that the low pH of glass ionomer cements while setting may contribute more to their antimicrobial properties than their fluoride-leaching capabilities.

Emilson^9^ reported that no antimicrobial compound with the exception of fluoride has been shown to be more effective in the prevention of dental caries than chlorhexidine. It has the property to inhibit the action of glucosyltransferase enzyme responsible for accumulation of bacteria on the tooth surface. Sugar transport and acid production in oral bacteria is also affected by chlorhexidine. In humans, the clinical efficiency of the combination of chlorhexidine and fluoride was tested by Luoma et al^10^ in children.

The results obtained display a very significant fall in the mutans streptococci count on days 1, 7, and 14 (Graph 3).

Türkün et al^11^ reported that antimicrobial activity was dependent upon the concentration of disinfectant added to glass ionomer cements and others indicated no dose-response effects.^12,13^ In our study, 5% chlorhexidine gluconate solution was added to glass ionomer liquid. The bacterial strain *S. mutans* was inhibited and the antimicrobial properties decreased with time may be because of decrease in available chlorhexidine.

The high concentrations of chlorhexidine in the mixture increases the time of antimicrobial effect on *S. mutans* but decreases the physical properties of the material.

The decrease in the physical properties of the gluconate form of chlorhexidine is related to the fact that it is a liquid and leaches out more rapidly than the powder or diacetate form of chlorhexidine.^13^ Furthermore, the stability of chlorhexidine solution is adversely affected by exposure to higher temperatures of light, which may happen during storage of glass ionomer liquid. On the contrary, the amount of chlorhexidine should be kept as low as possible, as the chlorhexidine does not contribute to the formation of the glass ionomer network, and therefore, high amounts of chlorhexidine would weaken the scaffold and compromise the physical properties of the antibacterial glass ionomer cements.^14^ In our study, 5% chlorhexidine gluconate solution was added to glass ionomer liquid 1 minute before preparing the specimens.^15^

In our study, the highest amount of antimicrobial activity with mean inhibitory zone was found in Fuji II with chlorhexidine gluconate group followed by Fuji IX with chlorhexidine gluconate, Fuji II without chlorhexidine gluconate and Fuji IX without chlorhexidine gluconate. These results coincided with findings reported by Frencken et al^16^ and Shashibhushan et al.^7^ Fuji IX contains high amounts of glass, immediately after mixing, it becomes highly viscous and in wet environment it shows increased surface hardness.

It is considered that release of limited amounts of chlorhexidine is due to viscosity and hardness of Fuji IX irrespective of its contents and solubility. Therefore, it can be concluded that chlorhexidine-containing glass ionomer cements showed a superior effect in inhibiting growth of microorganisms compared with conventional glass ionomer cements alone. Research is going on in developing glass ionomer cements with antibacterial effects by the addition of bactericides such as chlorhexidine.

## CONCLUSION

- All four groups of restorative materials showed antimicrobial properties against *S. mutans* and the properties decreased with time.
- The highest amount of antimicrobial activity with mean inhibitory zone was found in Fuji II with chlorhexidine gluconate followed by Fuji IX with chlorhexidine gluconate, Fuji II without chlo-rhexidine gluconate and Fuji IX without chlorhexidine gluconate.
- This study concludes that the addition of chlorhexidine gluconate to Fuji II and Fuji IX glass ionomer cements resulted in a restorative material that had increased antimicrobial properties over the conventional glass ionomer cements alone for *S. mutans.*
